# Neurotensin (NTS) and its receptor (NTSR1) causes EGFR, HER2 and HER3 over-expression and their autocrine/paracrine activation in lung tumors, confirming responsiveness to erlotinib

**DOI:** 10.18632/oncotarget.1633

**Published:** 2014-09-27

**Authors:** Mohamad Younes, Zherui Wu, Sandra Dupouy, Audrey Mansuet Lupo, Najat Mourra, Takashi Takahashi, Jean François Fléjou, Jean Trédaniel, Jean François Régnard, Diane Damotte, Marco Alifano, Patricia Forgez

**Affiliations:** ^1^ INSERM-UPMC U938, Hôpital Saint-Antoine, Paris, France; ^2^ INSERM UMR-S 1007, Université Paris Descartes, Paris, France; ^3^ Pathology Department, Université Paris Descartes, Hôpitaux Universitaires Cochin Hôtel-Dieu Broca, Paris, France; ^4^ Pathology Department Hôpital Saint-Antoine, Paris, France; ^5^ Division of Molecular Carcinogenesis, Center for Neurological Diseases and Cancer, Nagoya, Japan; ^6^ Thoracic Oncology Department, Hôpital Saint-Joseph and Université Paris Descartes, Paris France; ^7^ Thoracic Surgery Department, Université Paris Descartes, Hôpitaux Universitaires Cochin Hôtel-Dieu Broca, Paris, France

**Keywords:** Cancer growth and metastasis, neurotensin, EGFR, HER2, HER3, EGF like ligands

## Abstract

Alterations in the signaling pathways of epidermal growth factor receptors (HERs) are associated with tumor aggressiveness. Neurotensin (NTS) and its high affinity receptor (NTSR1) are up regulated in 60% of lung cancers. In a previous clinical study, NTSR1 overexpression was shown to predict a poor prognosis for 5 year overall survival in a selected population of stage I lung adenocarcinomas treated by surgery alone. In a second study, shown here, the frequent and high expression of NTSR1 was correlated with a pejorative prognosis in 389 patients with stage I to III lung adenocarcinoma, and was an independent prognosis marker.

Interactions between NTS and NTSR1 induce pro-oncogenic biological effects associated with neoplastic processes and tumor progression. Here we highlight the cellular mechanisms activated by Neurotensin (NTS) and its high affinity receptor (NTSR1) contributing to lung cancer cell aggressiveness. We show that the NTS autocrine and/or paracrine regulation causes EGFR, HER2, and HER3 over-expression and activation in lung tumor cells. The EGFR and HER3 autocrine activation is mediated by MMP1 activation and EGF “like” ligands (HB-EGF, Neuregulin 1) release. By establishing autocrine and/or paracrine NTS regulation, we show that tumor growth is modulated according to NTS expression, with a low growth rate in those tumors that do not express NTS. Accordingly, xenografted tumors expressing NTS and NTSR1 showed a positive response to erlotinib, whereas tumors void of NTSR1 expression had no detectable response. This is consistent with the presence of a NTS autocrine loop, leading to the sustained activation of EGFR and responsible for cancer aggressiveness.

We propose the use of NTS/NTSR1 tumor expression, as a biomarker for the use of EGFR tyrosine kinase inhibitors in patients lacking EGFR mutation.

## INTRODUCTION

Lung cancer is the leading cause of cancer-related deaths in western countries [1,2]. Despite advances in medical and surgical care, the 5-year overall survival of NSCLC remains poor (10 to 20%) [3,4]. The stage of the disease is the most useful parameter in deciding management and defining prognosis. However, survival is heterogeneous even within a single stage [3,5]. Therefore, the identification of biological parameters allowing characterization, and validation of tumor subsets possessing aggressive phenotypes, is an ongoing challenge for lung cancer biological studies. In recent years, significant progress has been made in understanding the molecular mechanisms of bronchial carcinogenesis. For example, recent success in new cancer therapies targeting epidermal growth factor receptors, or their downstream signalization, illustrated their decisive roles in tumor aggressiveness [6,7]. Malignant transformation of normal bronchial cells is a multistep process, characterized by the accumulation of sequential genetic or epigenetic alterations [8]. Some of these genetic alterations such as EGFR mutations, and ALK gene rearrangements offer opportunities to identify and characterize subpopulations of patients eligible for specific therapies [9,11].

In tumoral cells, EGFR, HER2, HER3, and HER4 functions can be abnormally exacerbated because of genetic defaults, protein over-expression, or over-activation of one or several of these receptors. In tumors, these regulations are not mutually exclusive and confer a large magnitude of oncogenic activities. Constitutive activation of HERs induces sustained proliferative signaling, and activates invasion and metastasis, both hallmarks of cancer [8]. Constitutive activation of HERs becomes installed with the overexpression of matrix metalloproteinases (MMPs) and the subsequent activation of EGF “like” ligands. Activation of the matrix metalloproteinases, MMPS and ADAMS, provokes the shedding of EGF “like” ligands [12], with autocrine or juxtacrine activation of EGFR and HER3, occurring with the shedding of HB-EGF, a specific ligand for EGFR, and neuregulin a specific ligand for HER3 [13]. Whereas, it is clear that this participates in the aggressive phenotype of the tumoral cells, it is difficult to target this cascade with therapeutic molecules because it encompasses many factors and effectors. Finding an upstream factor which could be pharmacologically targeted would be a more successful strategy.

NTS, a 13 amino acids peptide, is present and biologically active in the central nervous system and in periphery [14,15]. At the peripheral level, neurotensin is released by the endocrine cells (N) of the intestinal mucosa after meals and acts as an endocrine hormone involved in the postprandial regulation of the motor functions of the gastrointestinal tract [16]. The effects of NTS are mediated by three subtypes of receptor; NTSR1 and NTSR2 exhibit high (sub-nanomolar) and low (nanomolar) affinity for NTS, respectively, and belong to the family of G protein receptors. NTSR3 or gp/95/sortilin is a single transmembrane domain receptor [17].

Exogenous activation of NTSR1 leads to cell proliferation, survival, mobility and invasion in cancer cells from diverse origin [18,19]. These effects are the result from the activation of kinases and effectors, such as PKC, MAPK, FAK, RHO-GTPase, RAS and Scr [20,25]. The PKC activation may induce MAPK by direct stimulation of Raf-1, or by transactivation of the Epidermal Growth Factor Receptor (EGFR) [20,26,27]. The activation of MAPK via NTSR1 is mainly associated with uncontrolled cell growth, which aggravates the growth of tumors [28,29].

We have focused on the contribution of NTS/NTSR1 complex in breast and lung cancers. In breast cancer cells, NTSR1 up regulation was the result of beta catenin cellular and nuclear delocalization [30]. Both NTS and NTSR1 were expressed in 20 and 40 % of the tumor breast and lung tumors, respectively. NTSR1 high expression is a negative prognostic marker in a selected population of stage I lung adenocarcinomas, treated by surgery alone, and in ductal invasive carcinomas [31,32]. The removal of NTSR1 expression in both lung and breast cancer cells caused a reduction of tumor growth and metastasis, demonstrating the contribution of this complex in tumor progression in breast and lung cancers [31,33]. NTSR1 is a G protein coupled receptor endocytosed following NTS exposure. We have shown that intense and chronic NTS exposure lead to the sustained activation of NTSR1 signaling and NTS targeted genes [34,36]. These cellular conditions are similar to autocrine and paracrine exposition that would occur in human tumor cells since both factors are expressed in human cancers.

In this study, we highlight the contribution of autocrine and paracrine NTS regulation to lung cancer cell aggressiveness. We show that sustained stimulation of NTSR1 results in the activation of MMP1, the release of HB-EGF and NGR1 followed by EGFR, HER2 and HER3 overexpression and activation. This cascade results in an increase in the growth of experimental lung tumors.

## RESULTS

### The NTS/NTSR1 complex enhances cellular growth

**Figure 1:**
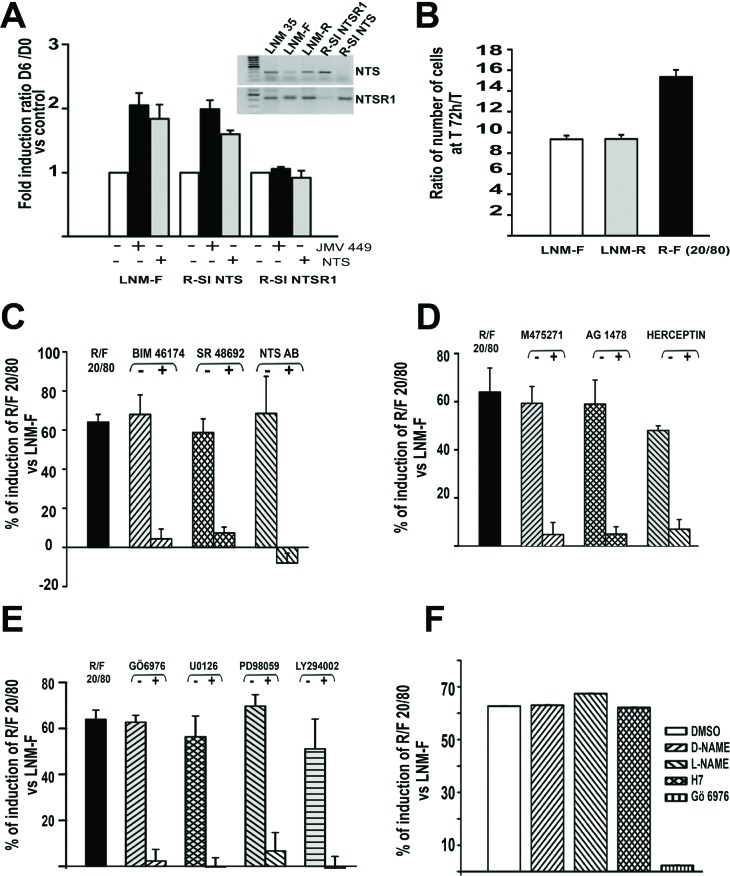
NTS autocrine and paracrine regulation enhanced cellular growth in human lung cancer cell lines **(A)** Influence of NTS exogenous treatment on lung cancer cell growth. LNM-F, R-SI NTS and R-SI NTSR1 were grown in media containing 0 % FCS at low concentration and treated every day with 10^-8^ M NTS or JMV 449 for 6 days. The ratio of the number of cells at Day 6/Day 0 was calculated. The result is expressed as the % of fold induction. *Inset*, NTS and NTSR1 transcripts analysis from a total of 200 ng of LNM-35, LNM-R, LNM-F, R-SI NTSR1 and R-SI NTS total RNA. **(B)** LNM-R and LNM-F were seeded alone or at the ratio of 20/80 LNM-R/LNM-F and grown in 0.1% FCS for 72h. The results are expressed as the ratio of the number of cells at 72h to T0 was calculated, and are the mean ± SEM of 7 independent experiments. **C to F**) LNM-R and LNM-F were seeded alone or at the ratio of 20/80 LNM-R/LNM-F and grown in 0.1% FCS for 72h, The ratio of the number of cells at 72h to T0 was calculated. The results are expressed as the percentage of the growth induction compared to LNM-F. Results are the mean ± SEM of 2 to 5 independent experiments. Cells were exposed to **(C)** DMSO, 10^-7^ M BIM 46174, 10^-6^ M SR 48692, 1/200 rabbit IgG or anti NTS antibody. **(D)** DMSO, 10^-7^M M475271, 10^-5^ M AG1478, PBS, or 50 μg/ml Herceptin. **(E)** 5×10^-6^ M Gö6976, 10^-6^ M U0126, 10^-6^ M PD98059, or 10^-7^ M LY294002. F) DMSO, 10^-5^ M D-NAME, 10^-5^ M L-NAME, 10^-5^ M H7 or 5 10^-6^ M Gö6976.

In previous studies, we showed that both NTS and NSTR1 are concomitantly expressed in human lung tumors. NTS actions, possibly occurring in tumor, are therefore mediated through autocrine and/or paracrine regulation [31]. In order to evaluate the contribution of NTS in the context of autocrine and/or paracrine regulation, we studied cellular subpopulations from the highly metastatic lung carcinoma cell line, LNM-35 [37]. LNM-R cells (expressing NTS and NTSR1) and LNM-F cells (expressing mainly NTSR1) were isolated from the parental LNM-35 cells and the observed phenotypes remained with cultured passages (Figure 1A inset).

We confirmed the differential expression of NTS in the two subclones by radioimmunoassay. The LNM-R culture media contained large amounts of NTS, which accumulated with time (75 to 625 fmol/ml), whereas the media of LNM-F cells contained 20 fold less NTS (Figure 1S A). NTSR1 immunocytochemistry experiments revealed a non-activated NTS/NTSR1 state in LNM-F cells, with NTSR1 localization at the cell surface. In contrast, a constitutively activated state of NTSR1 was found in LNM-R cells as revealed by the localization of NTSR1 in a peri-nuclear area (Figure 1S B) [36].

We first evaluated the contribution of NTS/NTSR1 complex on cellular growth on the LMN-R cells silenced for NTS or NTSR1. The clones were named R-SI NTS and R-SI NTSR1, respectively [31] (Figure 1A inset). Exogenous chronic treatment (48h) of R-SI NTS cells with NTS or a low degradable NTS agonist, JMV 449, induced a two fold increase in the cellular growth (Figure 1 A). In contrast, R-SI NTSR1 cells were not responsive, as expected, since the NTSR1 was silenced.

To analyze the autocrine/paracrine cooperativity of the NTS/NTSR1 complex, we created an *in vitro* model, by mixing LNM-F and LNM-R cell subpopulations. Cells were seeded at sub-confluency with a ratio of 20% of LNM-R and 80% of LNM-F, (R/F 20/80), and counted after 72h of culture. This proportion of the cell subpopulations was chosen because it is similar to the proportion of LNM-R and LNM-F cells in the parental cell line, LNM-35. We observed an increase of 60% in the number of cells of the mix R/F 20/80 compared to LNM-F or LNM-R culture alone (Figure 1B). Fluorescence activated cell sorting showed a higher proportion of cells in S phase and a smaller proportion in G1 phase, as compared to LNM-F cells cultured alone (Figure 1S C). To confirm the implication of NTSR1 in the observed growth induction in R/F 20/80, cells were exposed to BIM 46174 [38], an inhibitor of heterotrimeric G proteins, SR 48692 [39], a specific NTSR1 antagonist, and NTS neutralizing antibody. These compounds abolished the increase of tumor growth observed in the cell mixture R/F 20/80 (Figure 1C). A contribution of epidermal growth factor receptors (HERs) to induce NTS cellular growth was suggested by the abolishing effect of M475271, a Src kinase inhibitor, AG 1478, a specific inhibitor of EGFR, and herceptin (trastuzumab), an antibody specific to HER2, which abrogate the growth enhancement effect (Figure 1D). Chemical inhibitors confirmed the contribution of NTSR1 and HERs downstream pathways. Cellular growth amplification was abolished by a PKC inhibitor, Gö 6976, (Figure 1E), whereas the NO inhibitor, L-NAME, and the PKA inhibitors, H7, had no effect (Figure 1F). The effect was also abolished by MEK Inhibitors, U0126 and PD98059, and the phosphoinositide 3-kinases inhibitor, the LY294002 (Figure 1E).

### The NTS/NTSR1 complex enhances EGFR, HER2 and HER3 expression and activation

**Figure 2:**
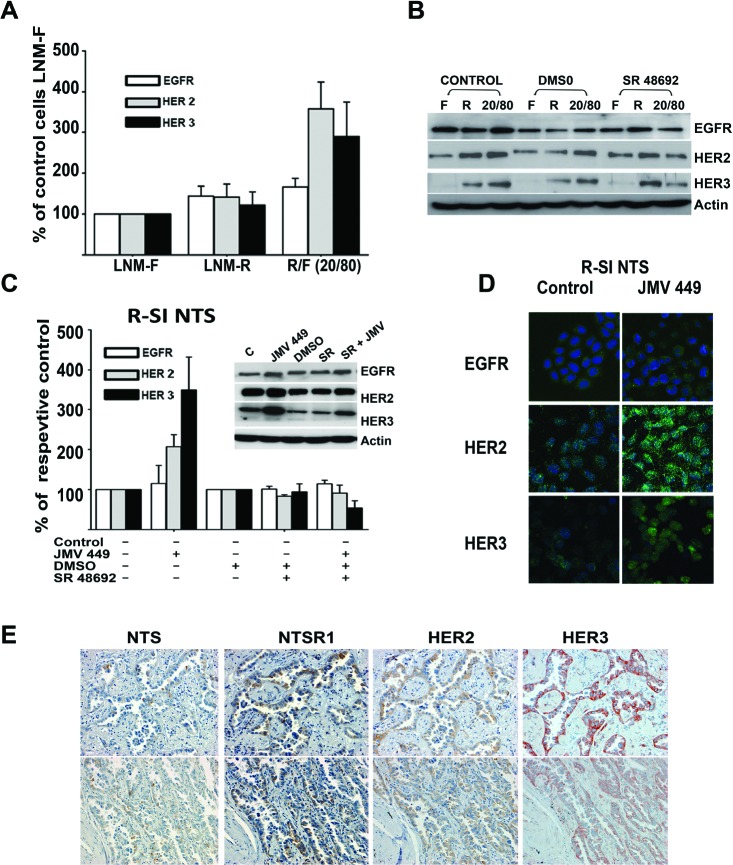
NTS regulation enhanced HER2, and HER3 basal expression in human lung cancer cell lines **(A)** The mixture of cells R/F 20/80 lung cancer cells cultured for 72h, with the histograms representing intensity-based quantification of Western blot bands of basal total protein, EGFR, HER2, and HER3, Values are expressed as the percentage of the control LNM-F cells (which are the population more representative of the mixture) and are the mean ± SEM of 5 to 8 independent experiments. **(B)** An example of western blot gel of LNM-F, LNM-R and the mixture LNM-F, LNM-R (20/80) cultured for 72h no treated or treated with DMSO or 5x10^-6^M SR 48692. The blots were revealed with EGFR, HER2 or HER3 antibodies. The actin shown is to the protein control for the HER3 Blot **(C)** Lung cancer cells R-SI NTS treated or not with 10^-7^M JMV 449, DMSO or 5x10^-6^M SR 48692 for 48h. The histograms represent intensity-based quantification of Western blot bands of basal total protein, EGFR, HER2, and HER3. Values are expressed as the percentage of the non-treated cells (control), and are the mean ± SEM of 3 to 6 independent experiments.. *Inset*, An example of western blot gel of R-SI NTS cells treated with 10^-7^M JMV449, DMSO or 5x10^-6^M SR 48692 for 48h. Western blot bands of basal total EGFR, HER2, and HER3 protein. **(D)** EGFR, HER2, and HER3 immunolabeling in R-SI NTS cells treated of not with 10^-7^ M JMV449 for 48 h. **(E)** Example of two restrictive areas from a patient with lung adenocarcinoma with a positives labeling for NTS, NTSR1, HER2, HER3.

The previous results highlighted a specific effect of NTS in oncogenic processes occurring through an interrelation between NTS/NTSR1 and receptor tyrosine kinase systems. We therefore measured the HERs cellular protein content in the mixture of R/F 20/80 cells cultured as previously described. An increase of HER2 and HER3 protein levels, and to a minor extent, EGFR protein levels was observed (Figure 2A). This effect was abolished by SR 48692 as shown on gel figure 2B. Surprisingly, similar mRNA levels were seen for the three receptors in LNM-R/LNM-F 20/80 as well as LNM-R and LNM-F cultured alone (Figure 2S). The accumulation of the HERs protein without transcriptional regulation suggests that the recycling and degradation of these receptors is altered by NTS/NTSR1 interaction. This is in line with our previous findings showing that sustained NTSR1 activation installs a state of permanent recycling of NTSR1, instead of agonist induced lysosomal degradation [36].

Western blot analysis of R-SI NTS cells exposed for 48h to exogenous NTS agonist also showed a marked increase of HER2 and HER3 protein content. These increases were totally abolished by SR 48692 treatment (Figure 2C and inset). No obvious changes was observed, by immunocytochemistry, in EGFR labeling in R-SI NTS cells, treated or not with JMV 449. In contrast, HER2 and HER3 staining were more intense at the membrane and in the cytosol of cell exposed to NTS agonist (Figure 2D). In both experiments, continued exposition to NTS in cells expressing NTSR1 induced the remodeling of HER2 and HER3 expression associated with more aggressive phenotype.

To explore if these mechanisms occur in human tumoral cells, we searched the consecutive slides from 27 specimens with lung adenocarcinoma for clusters of cells concomitantly labeled for NTS, NSTR1, HER2 and HER3. Concomitant expression was observed in restrictive areas of 19 specimens, and examples are shown in figure 2E. However, in 8 other specimens' concomitant overexpression could not be observed. These observations suggest the up regulation of HER2 and HER3 by NTS is specific of lung tumoral cells.

### NTS induced EGFR, HER2 and HER3 activation mediated by MMPs activation and EFG “like” ligand release

**Figure 3:**
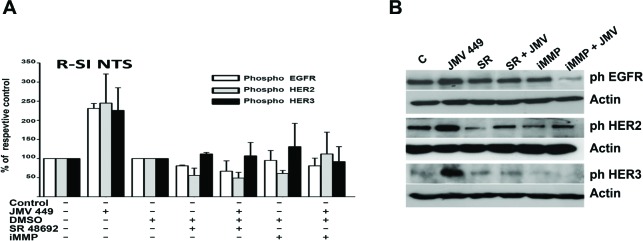
NTS regulation enhanced EGFR HER2, and HER3 activation in human lung cancer cell lines **(A)** Lung cancer cells R-SI NTS treated or not with 10^-7^M JMV 449, DMSO, 5x10^-6^M SR 48692 or 25 10^-9^M iMMP for 48h. The histograms representing intensity-based quantification of Western blot bands of phosphorylated protein, EGFR, HER2, and HER3. Values are expressed as the percentage of the non-treated cells (control), and are the mean ± SEM of 3 to 4 independent experiments. **(B)** An example of a western blot gel of R-SI NTS cells treated with 10^-7^M JMV449, DMSO, 5x10^-6^M SR 4869 or 25 x10^-9^M iMMP for 48h. Western blots bands of phosphorylated EGFR, HER2, and HER3 protein.

**Figure 4:**
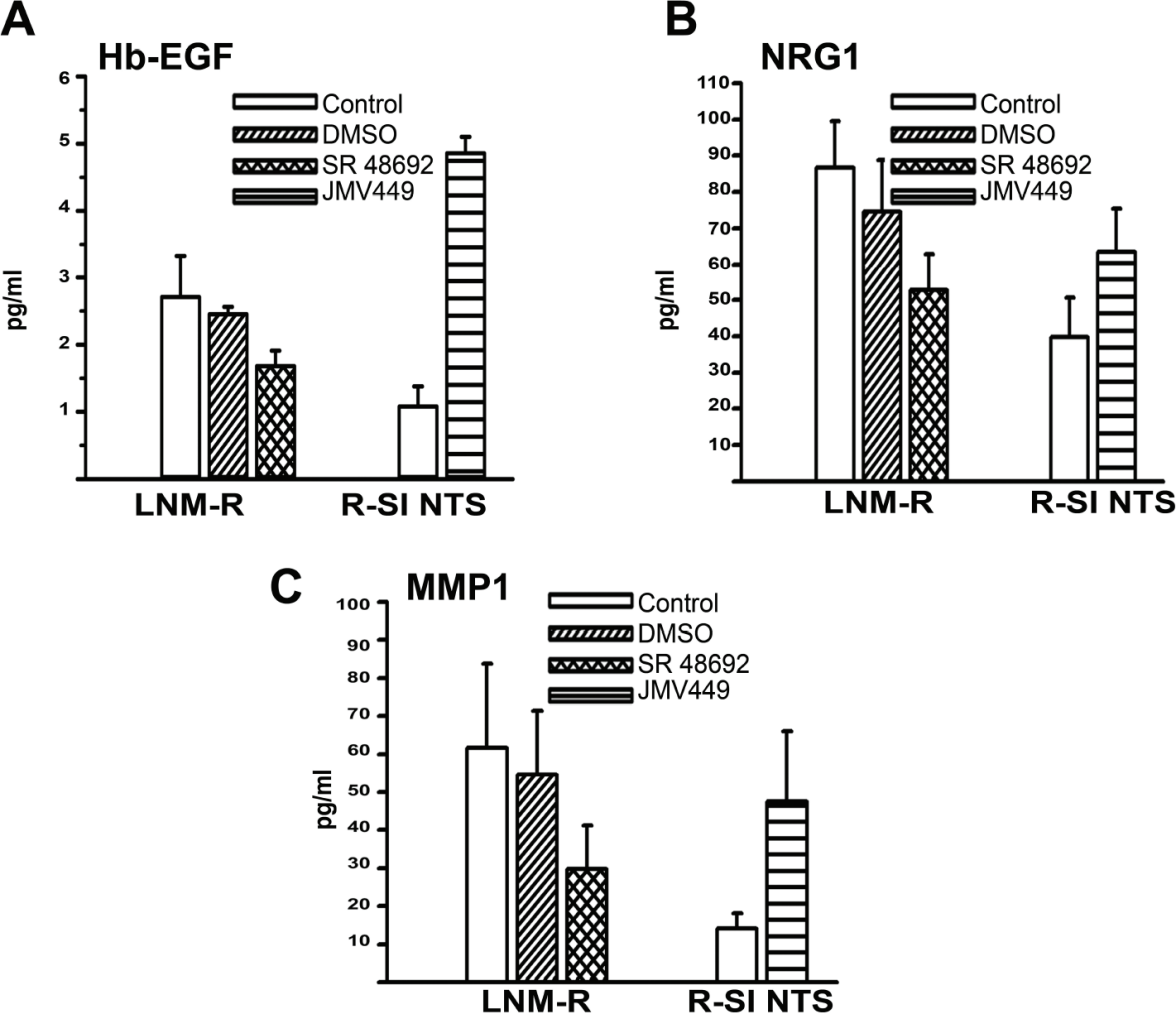
NTS autocrine and paracrine regulation activate EGF “like” ligands and MMP1 in lung cancer cell lines **(A)** Amount of Hb-EGF (pg/ml), assayed in 0% FCS culture media of LNM-R cells not treated, or treated for 48h with DMSO, 5x10^-6^M, SR 48692. R-SI NTS cells were treated or not for 24h with 10^-8^M JMV449. Using Paired t test p = 0.065 between DMSO and SR 48692 in LNM-R treated cells n=3; p=0.0015 between control and JMV 449 R-SI NTS treated cells n=3. **(B)** Amount of NRG1 (pg/ml) assay in 0% FCS culture media of LNM-R cells not treated or treated for 48h with DMSO, 5x10^-6^M SR 48692, or in R-SI NTS cells treated for 48h with 10^-8^M JMV449. Paired t test p = 0.04 between DMSO treated and SR 48692 LNM-R treated cells n=8; p=0.001 between control and JMV 449 R-SI NTS treated cells, n=7. **(C)** Amount of MMP1 (pg/ml) assay in 0% FCS culture media of LNM-R cells not treated or treated for 48h with DMSO, 5x10^-6^M SR 48692, or in R-SI NTS cells treated for 48h with 10^-8^M JMV449. Using Paired t test p = 0.03 between DMSO and SR 48692 LNM-R treated cells, n=4; p=0.036 between control and JMV 449 R-SI NTS treated cells, n=7.

In parallel, we observed a sustained activation states for all three receptors. R-SI NTS cells treated by JMV449 for 48h, showed an increase of 250% for the three receptors. This enhancement was completely abolished with treatment by SR 48692 and a metalloproteinase inhibitor, iMMP (Figure 3A and B). Metalloproteinases are known, through proteolysis process, to establish HERs autocrine activation with the shedding or activation of EGF “like” ligand at the cell membrane. We searched for an activation of EGF “like” ligands by NTS. We found a major increase in HB-EGF levels in R-SI NTS cells media treated with NTS agonist for 24h, and a decrease of HB-EGF cellular production when LNM-R were exposed to SR 48692 (Figure 4A). In this cell, EGFR autocrine regulation by HB-EGF would be enhanced by the release of HB-EGF under the influence of NTS. Similarly, neuregulin 1 (NRG1), a specific ligand for HER3, was found more intensively released when R-SI NTS cells were treated with NTS agonist. In LNM-R cells, the NTSR1 antagonist, SR48692 reduced the amount of NRG1 released in the culture media. Increased amounts of activated NRG1 sustained the hypothesis of HER3 autocrine regulation established under NTS exposure (figure 4B). In parallel, MMP1 was found to be released in the media of R-SI NTS cells treated with NTS agonist (Figure 4C). In cells bearing NTS autocrine regulation, MMP1 released was also decreased in the presence of SR 48692 (Figure 4C). Several matrix metalloproteases are regulated in NSCLC including MMP1, which is up regulated in both adenocarcinomas and squamous cell lung cancer [40].

### NTSR1 activation in experimental tumors

**Figure 5:**
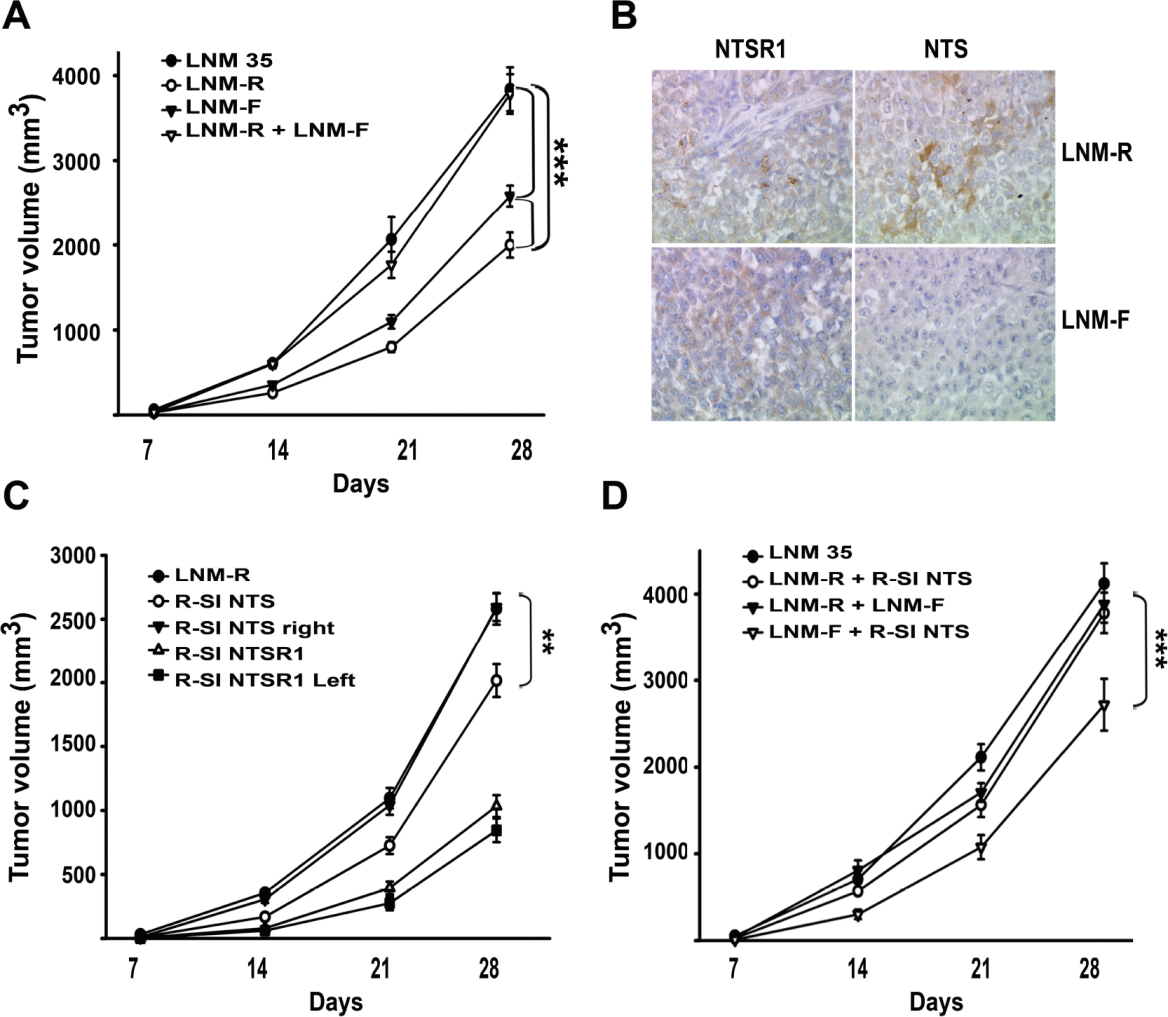
NTS/NTSR1 complex enhanced experimental tumor growth **(A)** Tumor growth generated by LNM35, LNM-R and LMN-F cells xenografted into nude mice. One million cells from LNM35, LNM-R, LNM-F, or a mixture of LNM-R and LNM-F (50/50) were subcutaneously injected in 24, 36, 34, or 12 nude mice, respectively. **(B)** Typical immunohistochemistry for NTSR1 (left) or NTS (right) for tumors generated from LNM-R (top) or LNM-F (bottom) cells. Significant differences at *** P < 0.001 or ** P < 0.01 using analysis of variance and Student-Neuman-Keuls test. **(C)** One million R-SI NTS cells were injected into the right mice flanks, and one million R-SI NTSR1 cells were injected into the left mice flanks of the same mouse (n=18). In a second set, one million LNM-R cells were injected only into the right mice flanks (n=36). **(D)** Tumor growth generated by mixture of cells expressing or not NTS One million LNM35 or a 50/50 mixture of R-SI NTS and LNM-R cells or a 50/50 mixture of LNM-R and LNM-F or a 50/50 mixture of R-SI NTS and LNM-F cells were injected in the right mice flanks, 28, 17, 11 and 14 mice were injected, respectively. For A, C and D Tumor volumes were measured every week. The ellipsoid formula (4/3 PI × (L/2 × l/2 × h/2)) was used to calculate the volume. Significant differences are shown at *** P < 0.001 and ** p < 0.01 using analysis of variance and Student-Neuman-Keuls test.

In order to apprehend the contribution of NTSR1 in lung tumorigenesis, we developed experimental tumors bearing NTS autocrine, and/or paracrine, or endocrine regulation. We established the growth rate of LNM35 tumor xenografts in the nude mice, in comparison with the two derived sub-clones, LNM-R (NTS+) and LNM-F (NTS-). As shown in figure 5A, LNM35 xenografts displayed the more drastic tumorigenesis profile with a final tumor volume of 4122 mm^3^. The sub-clones LNM-R, and LNM-F generated smaller tumors with a final volume of 2582 and 1858 mm^3^, respectively. The tumor size is 38 and 55% smaller than LNM35 when generated by LNM-R and LMN-F, respectively. The difference in the tumor growth rates between the parental cells and the two subclones suggests a positive cooperativity between these two cellular populations. To confirm this hypothesis, we mixed the two subclones at the same density before injecting into the mice. The same rate of tumor growth was then observed by injecting LNM35 cells (4122 mm^3^) or the LNM-F and LNM-R mixture (3782 mm^3^), as shown in figure 5A. The tumor weight observed at 28 days post-injection followed the same variation as the tumor volume (Figure 3S A). NTS and NTSR1 immunohistochemistry was performed on tumors. The presence of NTSR1 was seen in both LNM-R and LNM-F tumors (Figure 5B), but with a granular and irregular intensity of labeling. In order to better visualize NTS, we used an antibody against a NTS precursor, which detected the presence of NTS precursor in LMN-R and its absence in LNM-F tumors (Figure 5B).

We explored the effects of NTS systemic regulation on the tumor growth enhancement. R-SI NTS cells were injected subcutaneously into the right flank and R-SI NTSR1 cells in the left flank of the mice. Figure 5C shows that the R-SI NTS tumor xenografts reach the size and weight (Figure 3SB) of the tumors initiated by the corresponding LMN-R parental cells, whereas R-SI NTSR1 tumors remain at the same smaller size that was observed in mice bearing only R-SI NTSR1 xenografts, suggesting that the circulating NTS produced by the R-SI NTSR1 tumor enhanced the tumor growth of R-SI NTS xenografted into the other flank.

We evaluated the relative tumorigenic potential of the NTS autocrine and/or paracrine regulation. R-SI-NTS and LNM-R cells were mixed to generate xenografts bearing autocrine and paracrine NTS regulation as expressed in the original parental cells LNM35 and in the mixture LNM-R and LNM-F. Alternatively, we mixed cell lines not expressing NTS (R-SI NTS and LNM-F cells). When R-SI NTS and LNM-R cells mixture was injected in mice, the size of the tumor generated by this heterologous cell population (4122 mm^3^) was similar to tumors generated by the parental LNM35 cells (3885 mm^3^), and the mixture of LNM-R and LNM-F (3782 mm^3^) (Figure 5D) demonstrating that in all cases, the NTS autocrine regulation participates with paracrine regulation to strongly enhance tumor progression. In contrast, when a mixture of the R-SI NTS and LNM-F cells, not expressing NTS, was xenografted in nude mice, the tumor volume was globally 40% smaller than the xenografts bearing NTS autocrine and paracrine regulation (Figure 5D). Identical observations were made for the tumor weight (Figure 3S C). When the cells do not release NTS, there is no cooperation between cells, and tumor growth is slower. The overall conclusion of this series of experiments suggests that NTS participates in enhancing tumor growth *via* autocrine, paracrine and systemic pathways.

### Tumors expressing NTS/NTSR1 are responsive to EGFR inhibitors

We evaluated the therapeutic effects erlotinib, and metformin, on cells expressing both NTS and NTSR1 and cells none expressing NTSR1. Erlotinib is an EGFR specific tyrosine inhibitors currently proposed to patients with advanced NSCLC patients harboring EGFR-activating mutations [41]. Metformin is an antidiabetic drug, and has recently been proposed as a potential anticancer compound [42]. Metformin was shown to disrupt the crosstalk between insulin receptor and NTS receptor in pancreatic cancer cells [43]. Furthermore, in addition to inhibiting the mTOR pathway, metformin prevents ERK activation induced by NTS and insulin [44].

**Figure 6:**
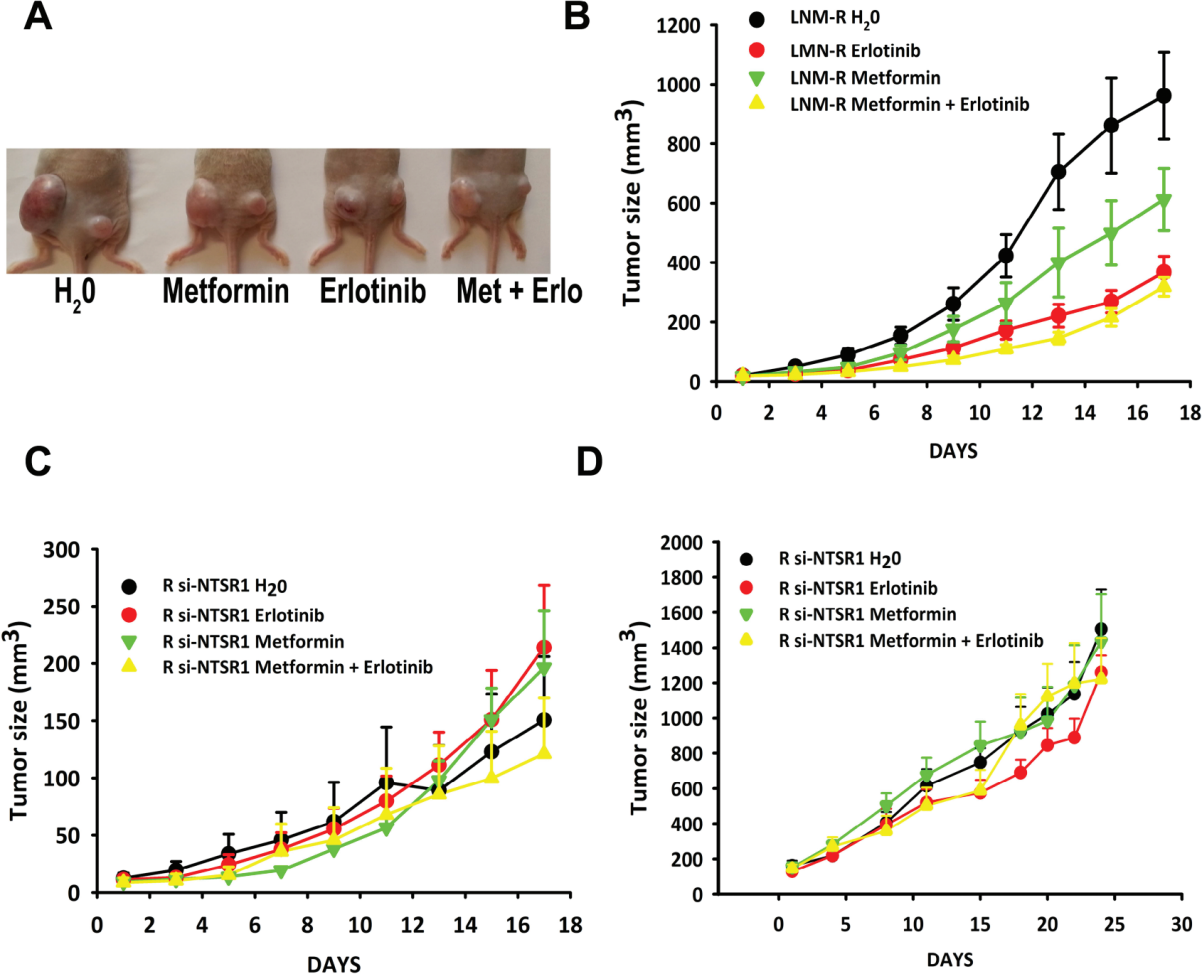
NTS/NTSR1 expressing tumors are the target for EGFR inhibitors treatment **(A)** LNM-R or R-SI NTSR1 cells (LNM-R expressing sh-RNA for NTSR1) were injected into the left and the right flank of the mice, respectively. Here is shown an example of a mouse from each group after 17 days of treatment. (**B** and **C**) Tumor growth generated by LNM-R cells (left flank) and R-SI NTSR1 cells (right flank) xenografted into nude mice and treated for 17 days with water, or 25 mg/kg erlotinib, or 200 mg/kg metformin, or both. At day one, 9 mice per group were randomized on LNM-R tumors size reaching approximately 20 mm^3^. **(D)** Tumor growth generated by R-SI NTSR1 cells xenografted into nude mice and treated for 24 days with water, or 25 mg/kg erlotinib, or 200 mg/kg metformin, or both. At day one, 10 mice per group were randomized on tumors size reaching approximately at 150 mm^3^.

Mice were xenografted with LNM-R cells expressing NTS and NTSR1 on the right side and with a derived clone R-SI NTSR1 [31], deleted for NTSR1 expression with a stable expression of sh-NSTR1 plasmid on the left side (Fig 6A). The LNM-R cells did not carry the following activating EGFR mutations: exon 19 deletion, exon 20 insertion, or exon 18 Q719A, Q719C, Q719S and exon 21 L858R, L861Q point mutations. As these cells are very aggressive, with a very high growth rate, we randomized the mice when the volumes of LNM-R tumors reached approximately 20 mm^3^. LNM-R tumors were sensitive to erlotinib and to a lesser extent to metformin. The final tumor volume after 17 days of treatment was 960.87 ± 146.19 mm^3^ for the control, 367.18 ± 53.55 mm^3^ for erlotinib (P= 0.0008 vs control), and 612.41 + 104.97 mm^3^ for metformin treated animals (P= 0.05 vs control), respectively. Used in co-treatment, metformin did not improve the response to erlotinib. The final volume was 318.23 ± 31.56 mm^3^ (Fig 6 B). The tumor doubling time was in agreement with the tumor volume, with 2.87 ± 0.13 days, 3.09 ± 0.07 days, 3.85 ± 0.24 days, and 4.03 ± 0.28 days for the control, metformin, erlotinib and metformin + erlotinib treated animals, respectively. The absence of a response of R-SI NTSR1 tumors to erlotinib or metformin, as shown in figure 6C, is consistent with the presence of a NTS autocrine loop, leading to the sustained activation of EGFR and responsible for cancer cell aggressiveness. To confirm the absence of an effect of erlotinib and/or metformin on R-SI NTSR1 tumors, another experiment were performed when the tumors reached the larger size of approximately 150 mm^3^. The tumors void of NTSR1 expression had no detectable response NTSR1 to metformin or erlotinib (Fig 6 D).

### Overexpression of NTSR1 in lung adenocarcinomas correlates with pejorative prognosis

A preliminary work of our team, suggested that the NTSR1 expression is a negative prognostic marker in a selected population of stage I lung adenocarcinoma treated by surgery alone [31]. We aimed therefore at assessing the prognostic significance of expression of NTSR1 in a population of consecutive patients with stage I-III NSCLC (all histotypes) referred to our institution for surgery. Firstly, we studied a population of consecutive patients operated on for NSCLC (all histotypes) between June 15, 2001 and June 14, 2002. Secondly, on the basis of initial results, only adenocarcinoma patients operated on between June 15, 2001 and December 31, 2005 were analyzed.

**Figure 7:**
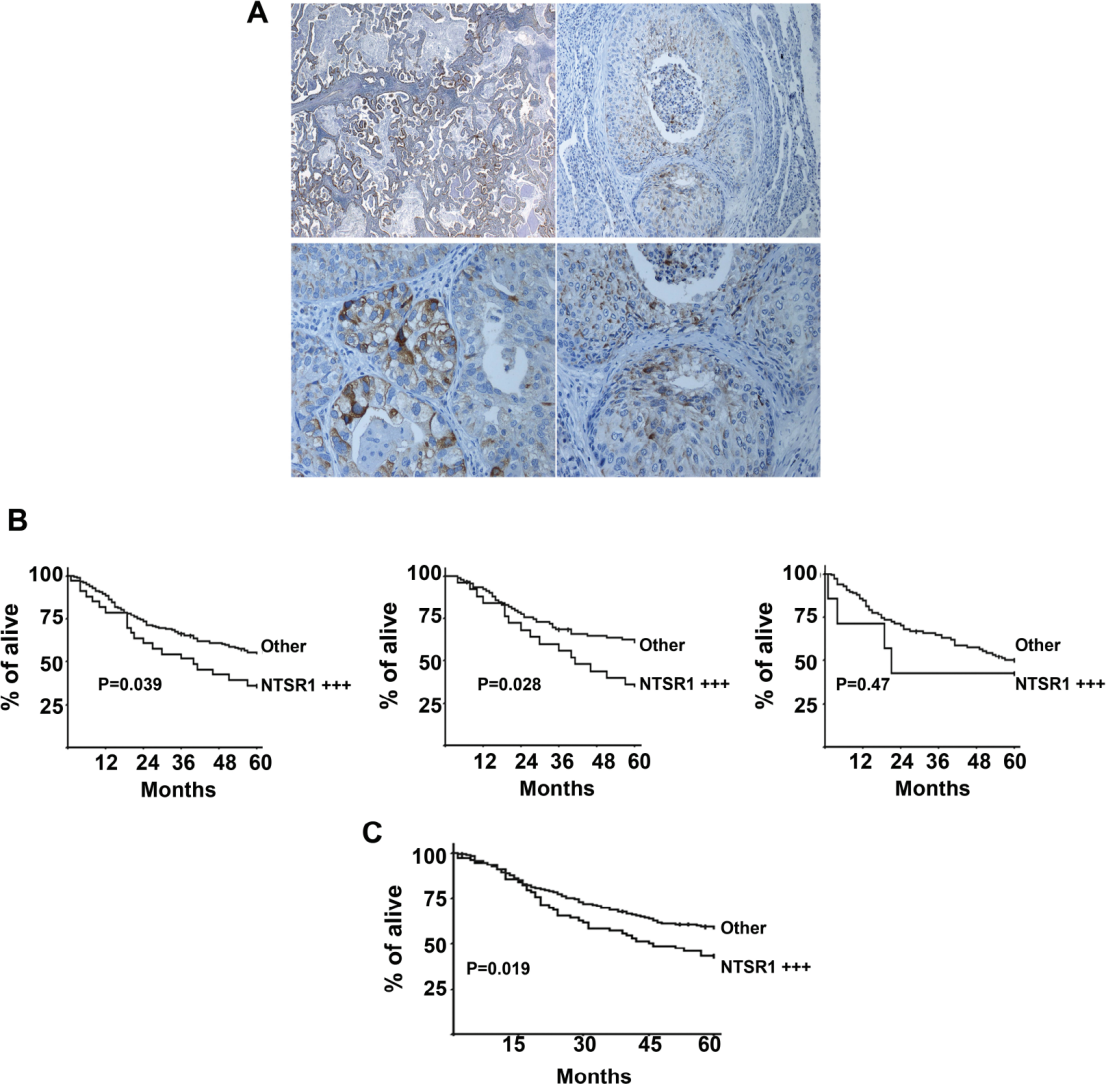
Immunohistochemistry of NTSR1, lung cancer tumors **(A)** NTSR1 Immunolabeling in patients with primary lung adenocarcinomas *(right)* top X 50, bottom X400) and Squamous Cell carcinomas *(left)*. top X100, bottom X200. **(B)** Overall survival of patients operated for NSCLC lung adenocarcinoma according to NTSR1 score. Semiquantitative immunohistochemistry evaluation of NTSR1: NTSR1 + + +: strongly positive expression (number of staining cells > 50% and the labeling intensity is high = score 2), other: the remaining patients (score 0 and 1). *left* Survival curve for the first cohort, *Center* Survival curve for lung adenocarcinomas from the first cohort, *right* Survival curve for SCC and LCC from the first cohort **(C)** Overall survival of patients operated for lung adenocarcinoma according to NTSR1 score. Semiquantitative immunohistochemistry evaluation of NTSR1: NTSR1 + + +: strongly positive expression (number of staining cells> 50% and the labeling intensity is high = score 2), other: the remaining patients (score 0 and 1).

In the first subpopulation (n=271; characteristics are described in table 1), presence of NTSR1 in more than 10% of staining cells was detected in 59 % of cases (160/271), but it was never detected in normal tissues adjacent to the tumor area. In the NTSR1 semi-quantitative evaluation, 111, 126, and 34 patients were scored as 0, 1 and 2, respectively. In the lung adenocarcinomas, NTSR1 staining of cancer cells was granular, intracellular, heterogeneous and rarely localized at the plasma membrane (figure 7A left). On the contrary, NTSR1 staining in the squamous carcinoma cells was often localized at the membrane level (figure 7A right). Interestingly, NTSR1 positive staining was not detected in lepidic carcinomas (formerly known as bronchioloalveolar) or even in the lepidic component of invasive adenocarcinomas.

**Table 1:** Clinical characteristics of patients

	First cohort	Second cohort	all adenocarcinomas
	n° 271 (%)	n° 270 (%)	n° 389 (%)
**Age mean-SD**	61.97 − (10,57)	60.77 − (11,01)	60.17 (10,84)
**Sex**			
men	228 (75.97)	183 (67.78)	272 (69.92)
women	43 (24.03)	87 (32.22)	117 (30.03)
**Tobacco history**			
Former smoker (stop > 2month)	152	110	163
Current smoker	101	72	124
Never smoker	16	37	52
Unknown	3	51	50
**Resection type**			
Lobectomy/bilobectomy	208 (77)	249 (92.16)	355 (91.3)
Pneumonectomy	63 (23)	21 (7.84)	34 (8.7)
**Stage**			
I	115 (42.5)	124 (46)	182 (46.8)
II	70 (25.8)	46 (17)	74 (19)
III	86 (31.7)	100 (37)	133 (34.2)
**Histological type** (n = 484)			
Adenocarcinoma (ADNK)	118	266	384
Bronchioloalveolar	1	4	5
Squamous cell carcinoma (SCC)	107	-	-
Large cell carcinoma	36	-	-
Mixed (ADNK+SCC)	6	-	-
Other (pleomorphic carcinomas)	3	-	-
**Intratumoral or peritumoral neoplasic vascular emboli**	121 (44.65)	104 (38.25)	158/389 (40.6)
**Intratumoral or peritumoral neoplasic lymphatic emboli**	69 (25.46)	101 (37.41)	131/389 (33.7)
**Perioperative Chemotherapy** (n = 267)	54/267 (20.22)	56/247 (22.67)	77/366 (21.04)
**Perioperative Radiotherapy** (n=267)	9/267 (3.37)	1/190 (7)	10/310 (3.23)
**Postoperative death**	20	10	12
**Follow-up in months**, mean -SD	41.4	42.4	42.6
**Lost at follow-up**	23	9	14
**Death during follow-up**, n (% of patients)	105/228 (46%)	120/261 (46%)	156/363 (42.9)
**NTSR1 score**			
0	111	142	163
1	126	78	150
2	34	50	76
**NTSR1**			
positive	160 (59)	128 (47)	226 (58)
negative	111(41)	142 (52)	163 (42)

NTSR1 score 1 was detected in 48 % of adenocarcinomas (57/119), 43% of squamous cell carcinomas (SCC) (46/107), and 39% of large cell carcinomas (LCC) (14/36). NTSR1 score 2 was detected in 22% of adenocarcinomas (26/119), 7% of SCC (8/ 107), and 3% (1/36) of LCC. NTSR1 score 2 was correlated with adenocarcinoma histological types (p = 0.013), but not with sex, age, smoking status, stage of disease and presence of vascular or lymphatic emboli. The prognostic significance of NTSR1 expression was assessed in 228 of the 271 patients, due to postoperative deaths (n=20) and loss at follow-up (n=23). NTSR1 score 2 was associated with poor 5-year overall survival as compared with NTSR1 scored 0 or 1 (36.5% [95% CI 22.27% − 53.5%]) versus 55.4% [95% CI 48.2% − 62.31%] respectively, p = 0.039) (Figure 7B Left). No difference was observed in survival between patients with NTSR1 score 0 and those with score 1. Among patients with adenocarcinomas, NTSR1 score 2 was significantly associated with worse 5-year overall survival as compared with NTSR1 score 0 or 1 (36.1% [95% CI 20.29% − 55.54%] versus 61.2% [95% CI 50.72% − 70.79%], p=0.028) (Figure 7B center). In contrast, among patients with either SCC or LCC, NTSR1 score did not predict survival (Figure 7B right).

The second populations focused only on adenocarcinoma subtypes. An additional 270 consecutive patients with adenocarcinoma was added (See patient baseline characteristics in table 1). Together, a total of 389 patients with adenocarcinomas were analyzed. The NTSR1 score 2 was observed in 19.5 % of patients (76/389). The correlation between NTSR1 and patient survival was determined on 363 of the 389 patients. The 5-year overall survival in this population was 55.3%. 5-year survival was 71.3%; 54.9%, 38.8%, 32.9% in patients with pT1, pT2, pT3 and pT4 tumors, respectively (p = 0.0000018). These figures were 65.1%, 50.7%, 34.7% for pN0, pN1, pN2 disease, respectively (p = 0.0000001). The NTSR1 score 2 was associated with worse 5-year overall survival as compared with NTSR1 score 0 and 1 (42.2% [32.42% − 54.74%] versus 58.5% [52.62% − 64.07%], p = 0.019) (Figure 7C). Multivariate analysis in all adenocarcinoma patients showed that pN (p=0.0000001), pT (p=0.00004) and NTSR1 score 2 (p=0.0069) were independent predictors of worse survival.

## DISCUSSION

Genetic defaults carried by tumors, represent specific biological markers which reveal altered regulatory pathways. The most well-known examples include the fusion genes (BCR-ABL and EML4-ALK), the activating mutations (EGFR, K-RAS, Scr, BRAF), and genomic amplification (HER2, MET). Accordingly, specific therapies employing EGFR and HER2 inhibitors or antibodies have been developed and were shown to improve the outcome of the disease. In parallel, cell signaling networks evolve with the accumulation of genetic and epigenetic alterations in connection with the local stroma and the immune system. Identification of contributing factors for tumor cell aggressiveness should enable to modulate tumor and disease progression.

Here we report that the NTS/NTSR1 complex contributed to tumor aggressiveness when it is abnormally over-expressed in tumors. The sustained activation of the NTS/NTSR1 complex generated cellular modifications resulting in the overexpression and continued stimulation of epidermal growth factor receptors. A similar result was detected using breast cancer cells. When injected into nude mice, we observed an increase in tumor growth and metastasis emergence from cells expressing NTS and NTSR1 as compared to cells expressing NTSR1 alone. The breast cells and the experimental tumors expressing NTS also displayed an increase in EGFR, HER2, and HER3 expression and activation. The latter effect was correlated with an increase of Hb-EGF, Neuregulin 2, and MMP9 [55].

Our experimental studies have shown that the NTS oncogenic action is boosted with a sustained NTSR1 state of activation. In human tumors, both NTS and NTSR1 are expressed in 40%, 60%, 65%, and 80 % of breast and lung cancers, mesotheliomas, and head and neck squamous cell carcinomas, respectively, suggesting that autocrine and/or paracrine NTS regulation occurs in tumors [32,45,46]. Sustained activation of NTSR1 induced the overexpression of the two receptors HER2, and HER3, as well as an autocrine activation of EGFR, HER2 and HER3. The transcriptional level of these three receptors was not altered by NTS, suggesting that a new equilibrium in the sequence synthesis-activation-degradation-recycling is therefore taking place in the cells under the influence of NTS. The higher expression of the HER2 isoform suggests that a larger proportion of dimers containing HER2 should be activated in the cell. This context was previously described in breast cancer cells with HER2 gene amplification. It was shown that the excess of HER2 intracellular domains impaired EGFR/HER2 endocytosis [47], by preventing the entry of activated EGFR into clathrin-coated vesicles and limiting the action of phosphatases and maintaining EGFR signaling [48]. In addition, it was also reported that under HER2 overexpression, the rate of lysosomal targeting was significantly reduced, and a rapid recycling of activated EGFR back to the cell surface occurred, as well decreased ligand dissociation from the EGFR [49]. The necessary threshold of HER2 expression levels to trigger these processes is not known.

Interestingly, we show in this report that NTS chronically activates the release of HB-EGF concomitantly with neuregulin 1. Consequently, both EGFR and HER3 autocrine activities are boosted and the tumoral aggressiveness is potentiated. The contribution of MMPs and EGF “like” ligands to carcinogenesis and cancer progression is well known. Therapeutical strategies targeting these factors have been largely attempted. However, these factors are also important for many pathway of the human metabolism, as well as and major physiological functions, such as healing, angiogenesis, and gonadogenesis [50], leading to toxic effects when antagonized. One approach is to specifically antagonize their effects in cancer cells and to target an upstream regulatory factor. In this context, the NTS/NTSR1 complex would appear to be convenient, because it is specifically over-expressed in tumors and its inhibition should only impair the function of these factors where they are deleterious. The validity of this concept was tested with the use of a specific NTSR1 antagonist, SR 48692, which significantly reduced all of the NTS induced oncogenic effects [18,51]. The use of new pharmacological molecules to antagonize or neutralize intense and constant NTS-NTSR1 activation should reduce tumor aggressiveness as tumoral cells bearing NTS and NTSR1 are susceptible to induce sustained activation of EGFR and HER3 concomitantly, as shown in figure 2E.

The LMN-R cells did not carry activating mutations in the tyrosine kinase (TK) domain of the EGFR gene that confers sensitivity to EGFR TKIs. The sustained activation of EGFR caused by a NTS/NTSR1 autocrine loop, mimics the effects seen by activating EGFR mutations. As expected, the tumors expressing the NTS/NTRS1 complex are responsive to erlotinib, an EGFR inhibitor given to patients with lung cancer bearing EGFR mutations [10]. In contrast, tumors void of NTSR1 expression and borne by the same mice were not responsive to erlotinib. No additional responses were seen with the concomitant treatment of metformin and erlotinib, confirming the hypothesis that the signaling events are in the same cascade.

We previously found that NTSR1 expression is associated with adenocarcinomas prognosis. This result was confirmed by multivariate analysis, which showed that among the available clinical and pathologic factors, the NTSR1 score 2, T, and N were independent predictors of worse prognosis. High expression of NTSR1 has been found to be associated to poor survival also in other cancers. Dupouy et al found that NTSR1 expression involving ≥ 80% tumor cells was associated with worse survival in breast cancer [51]. Similarly, in head and neck cancers, patients with high NTS and NTSR1 expression had a higher rate of distant metastasis [46]. Therefore, the prognostic role of the NTS system is probably correlated with its activation rate. In this context, the difference in staining between the different histological subtypes in our series explains the respective prognostic role of NTSR1. Within adenocarcinomas, NTSR1 staining was never detected in bronchioloalveolar subtypes or in the bronchioloalveolar component of mixed adenocarcinoma but it was often detected in its invasive compartment, suggesting a role favoring tumor invasion and migration. In SCC the staining was found primarily at the membrane as in the non-stimulated cells and was non associated with survival. *In vitro* studies have shown that NTS is capable of modulating the migratory ability of adherent cancer cells of different origins (colon, ductal pancreatic, head and neck squamous cell, breast). In addition, it has been showed that NTSR1 induces and enhances the invasive phenotype in prostate cancer cells (LNCaP) and HNSCC tumor cells. Involved mechanisms remain unclear but metalloproteinase are probably involved [46,52].

The NTS/NTSR1 complex could be used as a marker to identify subsets of human cancers, and thus make eligible new drugs, kinase inhibitors, or immunotherapy, targeting HERs protein or their downstream pathways. The clinical criteria used to propose these therapies are based on the detection of genetic defaults in the tumor (HER2 amplification, EGFR mutation). Nevertheless, it was also observed that other patient subsets could benefit from these therapies. The challenge is find a criteria to categorize them. For example, cells with neuregulin 1 high expression in association with HER3 autocrine activation and without HER2 amplification, are good responders to lapatinib or HER2 kinase inhibitors [53].

## CONCLUSION

Our findings implicate the NTS/NTSR1 complex as a contributor to cancer aggressiveness by enhancing concomitantly the expression and activation of three receptors EGFR, HER2, HER3. Presently, only EGFR mutated tumors are eligible to receive EGFR TKI, representing 10 % of all lung cancer patients [54]. From our findings, we propose that patients bearing this complex should be responders to kinase inhibitors, and that inhibition of NTS/NTSR1 complex should reduce the rate of tumor progression, providing a longer therapeutic window for the practitioners to treat their patients. Accordingly, our results indicate that an additional 20% of patient might benefit for these therapy.

## MATERIALS AND METHODS

### Cell culture procedures

The LNM35 cell line was sub-cloned by limiting dilution, after few days of culture, clones containing exclusively flat or rounded cells were saved and were named LNM-F for Flat, LNM-R for Rounded. All cells were grown at 37 °C, in a humidified atmosphere of 5% CO_2_.

### Cell proliferation assays

20 000 cells/well of lung cancer cells were seeded in 24-well culture plates. Medium was replaced by FCS-free medium in presence or absence of NTS or JMV449 10^−8^M. Cells were counted after 5 days of treatment with a particle count and size analyzer (Z1 Coulter Particle Counter, Beckman Coulter). For LNM-F/LNM-R (20/80 %) cell mixture: Cells were seeded in 48-well culture plates at a concentration of 40 000 cells/well, media containing 10% FCS. Media is changed 24h after for a media containing 0.1% FCS cells are counted after 48 hours.

### Western blots

2×10^6^ cells were grown for 72h then serum-starved for 48h in a phenol red-free medium in presence or absence of different concentrations of 5×10^−6^ M SR 48692 and 25×10^−9^M MMP inhibitor (Calbiochem), and lysed (20 mM Tris pH 8.0, 150 mM NaCl, 5 mM MgCl2, 0,5 % NP40, 0.5 % glycerol, 1 mM PMSF, protease and phosphatase inhibitor cocktail) at 4°C for 30 min. Primary antibodies were incubated overnight at 4°C according to the manufacturer's instructions. Total anti-EGFR (1:500), anti-phospho-EGFR (1:500), anti-phospho-HER2 (1:500), anti-HER3 (1:2000), anti-phospho-HER3 (1:1000) were from Cell Signaling Technology. Total anti-HER2 (1:2000) was purchased from Neomarkers and anti-βactin (1:50000) from Sigma. Secondary anti-rabbit (Santa Cruz Biotechnology) or anti-mouse (Sigma) antibodies, conjugated to HRP, were used at 1:2000 dilutions for 1h at room temperature and visualised by enhanced chemiluminescence (GE Healthcare).

### Immunofluorescence assays

Cells were seeded on 12 mm-diameter glass slides for 24 hours, fixed in 5 % paraformaldehyde for 1 hour at room temperature, permeabilized in PBS /0.5 % Triton X-100 for 30 min and saturated for 20 min in PBS+ (1:100 (m/v) BSA, 1:250 (v/v) cold fish skin gelatin in PBS 1X, pH 8.0). Cells were then incubated overnight at 4 °C with the primary antibody diluted to 1:100 in PBS 0.1 % Triton X-100. NTS immunoreactivity was detected using a rabbit polyclonal anti-NTS immunoglobulin (NA1230, Tebu-Bio) and NTSR1 with a goat polyclonal antibody directed against the human COOH terminus of the receptor (C20, Santa Cruz Biotechnology). Slides were incubated for 1 hour with a fluorescent secondary antibody (1:100) : a cyanin3 anti-rabbit immunoglobulin or a FITC-coupled anti-rabbit or goat immunoglobulin (Jackson ImmunoResearch). Nuclei were counterstained for 5 min with DAPI 1:50000.

### Tumor xenografts

Lung cancer cell xenografts were initiated in nude mice by subcutaneous injection of 10^6^ cells of LNM35, LNM-F, LNM-R, or derivative cell clones. For tumors generated from a cell mixture, 10^6^ cells from each clone were plated together 72 hours prior to injection. Four to six series were performed; each series included 5-8 mice. All procedures were in accordance with the “Guide of the Care and Use of laboratory Animals”.

For drug treatments, 10^6^ of LNM-R or R-SI NTSR1 cells (LNM-R expressing sh-RNA for NTSR1) were injected into nude mice by subcutaneous injection, R-SI NTSR1 cells in the right flank and LNM-R cells in the left flank. 7 days after injection, 4 groups of 9 mice were randomized on the size of LNM-R as follows: 19.79 ± 3.00 mm^3^ for control group, 18.66 ± 2.21 mm^3^ for erlotinib group, 16.82 ± 3.32 mm^3^ for metformin group and 18.82 ± 3.00 mm^3^ for metformin and elotinib group. Mice were treated for 17 days per os, with water, or 25 mg/kg erlotinib, or 200 mg/kg metformin or both. A second experiment was performed 15 days after injection of R-SI NTSR1 cells. Four groups of 10 mice were randomized as follows: 161.37 ± 29.13 mm^3^ for control group, 129.19 ± 20.89 mm^3^ for erlotinib group, 152.76 ± 27.86 mm^3^ for metformin group and 145.30 ± 23.4 mm^3^ for metformin and erlotinib group. Mice were treated for 24 days per os, with water, or 25 mg/kg erlotinib, or 200 mg/kg metformin, or both.

### Patients and tissue specimens for NTSR1 immunohistochemistry

A two-step procedure was followed. Firstly, we studied a population of consecutive patients operated on for NSCLC (all histotypes, including adenocarcinoma) in the Thoracic Surgery Dpt of the Hôtel-Dieu Hospital, Paris, France between June 15, 2001 and June 14, 2002. Secondly, on the basis of initial results, only adenocarcinoma patients operated on between June 15, 2001 and December 31, 2005 were analyzed. Patient characteristics, treatment procedures, and short-term and long-term outcomes were retrospectively collected using a standardized case report form. Furthermore, a centralized pathological blind revision of the samples was performed by two expert pathologists (D.D., A.L.). In this revision, histologic subtype was determined on the basis of the new International Association for the Study of Lung Cancer/American Thoracic Society/European Respiratory Society classification.

Adjuvant radiotherapy or chemotherapy was performed under the care of referring physicians, so no uniform protocol was employed. Long-term outcome was assessed by direct telephone interviews with patient or family (in case of deceased patients). When no clinical follow-up was available, information on vital status was obtained through the municipality of birth of the patient. Informed consent was obtained from all patients. The research was conducted according to recommendations outlined in the Helsinki declaration. Institutional Review Board approval was obtained (CPP Ile de France II, 2012).

### Immunohistochemistry

Procedure is detailed in SI. For all cases histologic slides of primary tumors were obtained from paraffin wax embedded tissues. Standard H&E staining was used to ensure the tumoral character of the specimen. Deparaffinized tissue sections (4 μm) were incubated at 4°C overnight with primary antibody included anti-NTS (1:200, SC-20806, Santa Cruz biotechnology®), anti-NTSR1 (1:100; C-20, Santa Cruz Biotechnology®) and anti-ErbB3 (1:50, NCL-c-erbB-3, Novocastra™), and anti-ErbB2 (1:400, A0485, Dako) was incubated at room temperature for 30 minutes.

For prognosis evaluation, all specimens were scored by an anatomopathologist with special interest in pulmonary pathology (DD). NTSR1 staining of cancer cells was scored as positive in the presence of staining cells > 10 %. Semi-quantitative evaluation was also performed: 0: no staining; 2: more than 50% of tumor cell showing a positive stain of high intensity; 1: intermediate cases.

### Statistical analysis

Statistical analysis was carried out using test student T test or Student-Newman-Keuls Multiple Comparisons Test : ***P<0.001,**P<0.01, and *P<0.05. For human studies, data processing and analysis were performed with the statistical software system SEM (SILEX Development, Mireffleurs, France). Correlations were carried out by the Spearman rank correlation or H-test, as appropriate. Survival analysis was carried out by the Kaplan-Meier method, and univariate comparisons of curves were performed using log rank tests. Risk factors associated with outcomes in univariate analysis with a p value <0.05 were entered into a multivariate Cox model analysis, to identify independent predictors of survival. A p value of less than 0.05 was considered significant.

## 

### Additional Methods

In order to confirm that both cell types were from the same origin, microsatellite analysis was performed using D17S250 and D17S513, which showed identical patterns for LNM-R, LNM-F and LNM35 (data not shown).

### RT-PCR and quantitative PCR

1μg of total RNA was subjected to reverse transcription, during 1 hour at 37 °C, using 1 μg of nonspecific hexameric random primers dN, 1mM dNTP, 10 mM dithiothreitol, 24 units RNaseOUT and 200 units of M-MLV-RT enzyme (Invitrogen). The PCR amplification was performed on 1:10 (v/v) of the 1:10-diluted reverse transcription reaction using 0.2 mM dNTP, 2.5 mM MgCl_2_ and 1 unit of Thermostart Taq DNA polymerase (Thermo Scientific), and 25 pmol of each specific primer :

NTS (5'-CAGCTCCTGGAGTCTGTGCT-3' and 5'-GAGTATGTAGGGCCTTCTGGG-3')
NTSR1 (-5'-CGTGGAGCTGTACAACTTCA-3 ' and 5'-CAGCCAGCAGACCACAAAGG-3)
HER3 (5'-ATGGGGAACCTTGAGATTGTGCT-3' and 5'-ACAGCTTCTGCCATTGTCCT-3')
EGFR (5'-TTTCGATACCCAGGACCAAGCCACAGCAGC-3' and 5' AATATTCTTGCTGGATGCGTTTCTGTA-3')
HER2 (5'-GTGCTAGACAATGGAGACC-3' and 5'-CACAAAATCGTGTCCTGGTAGC-3')
18S (5'-AGGAATTGACGGAAGGGCAC-3' and 5'-GTGCAGCCCCGGACATCTAAG-3')
36B4 (5'-GTGCAGCCCCGGACATCTAAG-3' and 5'-GATTGGCTACCCAACTGTTG-3')

Semi-quantitative amplification was performed in a DNA thermal cycler 9700 (Perkin Elmer Applied Biosystem), and Maxima SYBRGreen qPCR Master Mix (Fermentas) in a Mx3000P qPCR system (Stratagene) was used for quantitative PCR.

### NTS Radioimmunoassay (RIA)

RIA was performed on culture media from one million cells were grown in 60 mm^2^ Petri dishes for 24, 48, or 72 hours. RIA was performed as previously described Scarceriaux V et al Endocrinology 1995;136:2554-60, and detailed in Alifano M et al Clin Cancer Res 2010;16:4401-10.

### Flow cytometry

The assessment of the G1 and S phases of cell cycle on LNM-R, LNM-F and LNM-F/LNM-R mixtures were conducted using IP tests. 40 000 cells were seeded in 48-wells culture plates in complete medium. After 24 hours, medium was replaced by 0.1% FCS-counting medium. The cells were pelleted, 500 μL of 1nM IP was added to solubilize the pellet and incubated at 37°C during 10 min.

### Immunohistochemistry

Deparaffinized TMA sections (4 μm) were subjected to heat-induced epitope retrieval in citrate buffer (pH 6.0). The sections were labeled for the target proteins using the avidin-biotin-peroxidase complex method. The slides were incubated at 4°C overnight with primary antibody included anti-NTS (1:200, SC-20806, Santa Cruz biotechnology®) and anti-ErbB3 (1:50, NCL-c-erbB-3, Novocastra™), anti-NTSR1 (1:100; C20, Santa CruzBiotechnology®) and anti-ErbB2 (1:400, A0485, Dako) were incubated at room temperature for 1 hour and 30 minutes respectively. These slides were then incubated with appropriate biotinylated secondary antibodies, NTS (Trekkie Biotinylates rabbit link, Biocare medical®), NTSR1 (Biotinylated anti-goat IgG, Vector laboratories, Inc), ErbB3 (Trekkie Biotinylates mouse link, Biocare medical®). The antigen-antibody complex was revealed with avidin-biotinperoxidase complex, according to the manufacturer's instructions, NTSR1 (Vectastain ABC Kit, Vector laboratories, Inc.), NTS and ErbB3 (Trekavidin- HPR label, Biocare medical®). ErbB2 was biotinylated and revealed with the NovoLink™ Polymer Detection System (Leica®). NTSR1 and ErbB2 staining were done with diamino-benzidine tetrahydrochlorid, NTS and ErbB3 were done with aminoethyl carbazole. All slides were counterstained with hematoxylin.

For prognosis evaluation, NTSR1 immunoreactivity was detected using a specific antibody against the carboxy terminus of NTSR1 (1:100; C-20, Santa Cruz Biotechnology). These sections were then incubated with biotinylated secondary antibody (1:100; Vector laboratories, Inc). The antigen-antibody complex was revealed with avidin-biotinperoxidase complex, according to the manufacturer's instructions for the Vectastain ABC Kit (Vector laboratories, Inc.). Staining was done with diamino-benzidine tetrahydrochlorid. All slides were counterstained with hematoxylin.

## SUPPLEMENTARY FIGURES AND METHODS


