# Supplementary material for: Neurotensin (NTS) and its receptor (NTSR1) causes EGFR, HER2 and HER3 over-expression and their autocrine/paracrine activation in lung tumors, confirming responsiveness to erlotinib
Source: Oncotarget. 2014 Sep 27;5(18):8252–69. doi: 10.18632/oncotarget.1633 (PMC4226681; doi:10.18632/oncotarget.1633)
Supplement: Supplementary file 1 [file oncotarget-05-8252-s001.pdf]

## SUPPLEMENTARY FIGURES AND METHODS

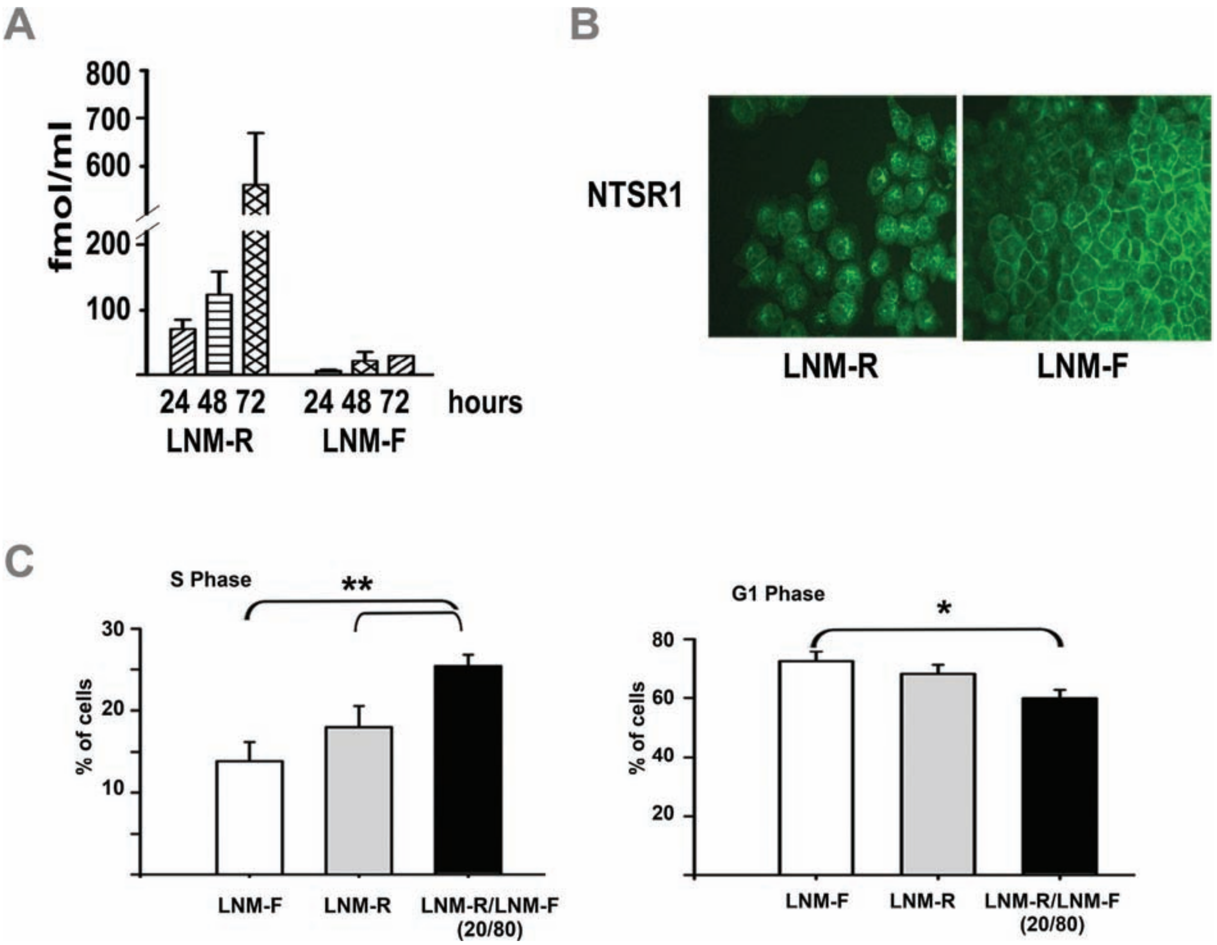


**Figure S1: Lung cancer cells characterization.** **(A)** NTS radioimmunoassay performed on cellular media of $10^6$ LMN-R or LNM-F cells grown for 24, 48, or 72h. Each experiment was performed 3 to 5 times in duplicate. **(B)** Autocrine regulation was demonstrated in LNM-R cells with NTSR1 immunocytochemistry. In LNM-F cells, NTSR1 is localized at the cell surface, demonstrating the naive status of the cells. In contrast, in LNM-R cells, NTSR1 is located in a peri-nuclear area, suggesting an intense internalization of the receptor. **(C)** Cell proliferation was measured by fluorescence activated cell sorting, in LNM-R and LNM-F seeded alone or seeded at the ratio of 20/80 % LNM-R/LNM-F. Left and right represents the % of in S and G1 phase, respectively.

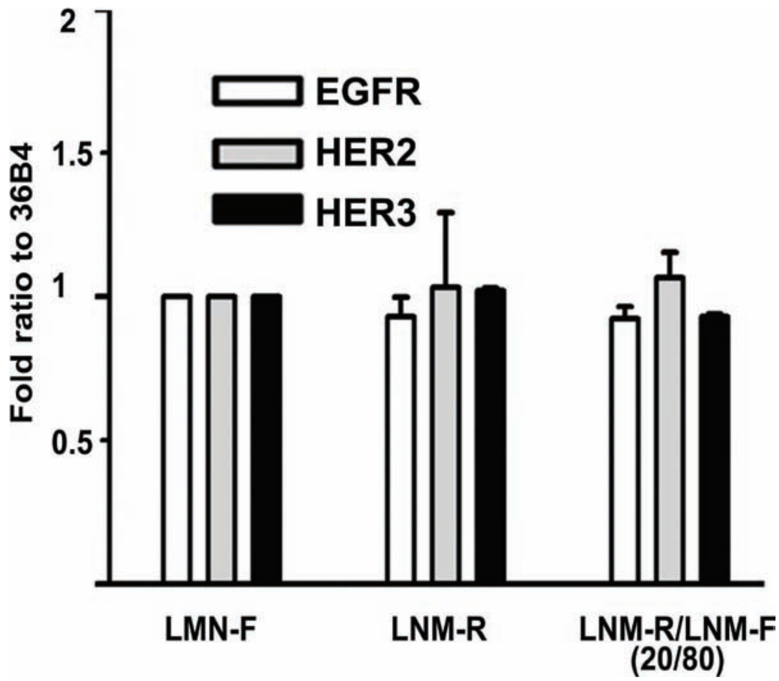


**Figure S2: EGFR, HER2, and HER3 Q PCR performed on LNM-F, LNM-R and the mixture (R/F 20/80).**

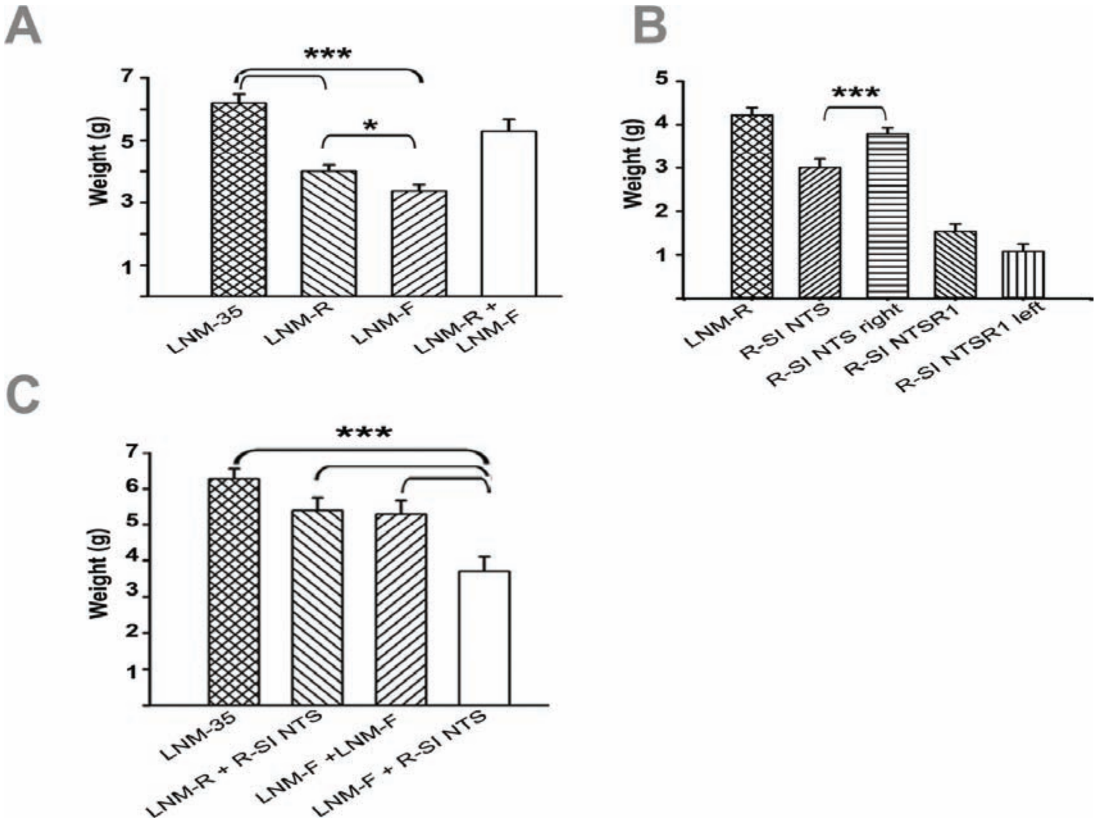


**Figure S3: NTS/NTSR1 complex enhances experimental tumor weight. (A)** Tumor weights generated by LNM35, LNM-R and LMN-F cells xenografted into nude mice. One million cells from LNM35, LNM-R, LNM-F, or a mixture of LNM-R and LNM-F (50/50) were subcutaneously injected in 24, 36, 34, or 12 nude mice, respectively. **(B)** NTS endocrine regulation and enhancement of tumor weight. One million R-SI NTS cells were injected into the right mice flanks, and one million R-SI NTSR1 cells were injected into the left mice flanks of the same mouse (n=18). In a second set, one million LNM-R cells were injected only into the right mice flanks (n=36). **(C)** Tumor weights generated by mixture of cells expressing or not NTS. One million LNM35 or a 50/50 mixture of R-SI NTS and LNM-R cells or a 50/50 mixture of LNM-R and LNM-F or a 50/50 mixture of R-SI NTS and LNM-F cells were injected in the right mice flanks, 28, 17, 11, and 14 mice were injected, respectively.
